# Identification of Germline Genetic Variants that Increase Prostate Cancer Risk and Influence Development of Aggressive Disease

**DOI:** 10.3390/cancers13040760

**Published:** 2021-02-12

**Authors:** Edward J. Saunders, Zsofia Kote-Jarai, Rosalind A. Eeles

**Affiliations:** 1 The Institute of Cancer Research, London SM2 5NG, UK; Zsofia.Kote-Jarai@icr.ac.uk (Z.K.-J.); Ros.Eeles@icr.ac.uk (R.A.E.); 2 Royal Marsden NHS Foundation Trust, London SW3 6JJ, UK

**Keywords:** prostate cancer, aggressive prostate cancer, prostate cancer susceptibility, prostate cancer genetics, genome-wide association studies, massively parallel sequencing studies

## Abstract

**Simple Summary:**

The potential importance of germline genetic variation for identifying men at increased risk of prostate cancer has become increasingly recognised in recent years. We present an extensive review of the major developments in the identification of genetic loci, genes and individual variants associated with greater risk of prostate cancer, and what is currently known regarding whether these heritable prostate cancer risk factors can also inform likelihood of experiencing clinically significant rather than indolent forms of the disease. We finally discuss how these research discoveries might serve to inform clinical germline genetic testing guidelines and treatment options for prostate cancer in the future.

**Abstract:**

Prostate cancer (PrCa) is a heterogeneous disease, which presents in individual patients across a diverse phenotypic spectrum ranging from indolent to fatal forms. No robust biomarkers are currently available to enable routine screening for PrCa or to distinguish clinically significant forms, therefore late stage identification of advanced disease and overdiagnosis plus overtreatment of insignificant disease both remain areas of concern in healthcare provision. PrCa has a substantial heritable component, and technological advances since the completion of the Human Genome Project have facilitated improved identification of inherited genetic factors influencing susceptibility to development of the disease within families and populations. These genetic markers hold promise to enable improved understanding of the biological mechanisms underpinning PrCa development, facilitate genetically informed PrCa screening programmes and guide appropriate treatment provision. However, insight remains largely lacking regarding many aspects of their manifestation; especially in relation to genes associated with aggressive phenotypes, risk factors in non-European populations and appropriate approaches to enable accurate stratification of higher and lower risk individuals. This review discusses the methodology used in the elucidation of genetic loci, genes and individual causal variants responsible for modulating PrCa susceptibility; the current state of understanding of the allelic spectrum contributing to PrCa risk; and prospective future translational applications of these discoveries in the developing eras of genomics and personalised medicine.

## 1. Introduction

Prostate cancer (PrCa) is the most frequently diagnosed cancer in males in Europe and North America and second most common worldwide, with over 1.4 million diagnoses recorded in 2020 [1,2]. Although a majority of patients present with indolent, slow developing disease, PrCa remains a substantial cause of mortality with over 375,000 deaths recorded worldwide in 2020. The five-year cause specific survival rate for men diagnosed with localised or regional PrCa is effectively 100%, however this deteriorates to only 30.1% in men with metastatic disease at the point of diagnosis [3,4].

Although several promising molecular and genomic biomarkers for PrCa diagnosis or management have been identified in recent years [5], prostate-specific antigen (PSA) remains the only biomarker to have been widely employed for PrCa detection to date. The ability of PSA to discriminate high-risk disease is however poor and it is therefore no longer widely advocated as a systematic screening tool [6]. Although modest evidence linking smoking [7] and possibly body mass index [8,9] to poorer prognosis in PrCa patients has been presented, few clear, modifiable lifestyle risk factors have been established for either disease development or progression. In the past decade, substantial progress has been made towards identifying heritable PrCa risk factors; however, the ability to discriminate patients or healthy men at greater risk of dying from PrCa remains modest. The identification of genetic risk factors predisposing towards more advanced clinical presentation of PrCa and more rapid disease progression would have the potential to facilitate targeted screening of individuals at higher risk of death from PrCa whilst concurrently reducing overtreatment of men with lower risk disease, or to inform decision making in treatment pathways [10].

No consistent, consensus definition of aggressive PrCa has thus far been adopted within the research or clinical settings, which can hinder comparability of studies to identify risk factors and uniformity of clinical treatment application. Prior to the advent of PSA testing, aggressive PrCa had been considered to encompass only cancers which had advanced beyond the prostate itself [11]. More recently, due to increasing diagnosis of men at younger ages who are presenting at earlier timepoints within their disease course, the definition of aggressive PrCa has routinely been expanded to encompass also men that have localised disease coupled with indicators reflective of higher risks of future progression to lethal phenotype. These updated classifications of aggressive PrCa may consider varying combinations of Gleason score (typically one of Gleason score ≥7, Gleason score ≥8 or Gleason grade group ≥3), T stage (generally either T4 or ≥T3), nodal invasion, metastatic spread, extreme PSA measurements, young age at diagnosis (generally defined as diagnosis at age < or ≤55 years, with ages 60 or 65 also frequently used as cut-offs), and death from PrCa. A recent analysis considering the sensitivity and positive predictive value of different definitions of aggressive disease with respect to discriminating patients that experience death from PrCa within 10 years of diagnosis has proposed the adoption of a standardised definition of aggressive PrCa in etiological research as being any one or more of stage T4 or N1 or M1 or Gleason score ≥8 disease at diagnosis [12].

## 2. Evidence of a Genetic Basis for Prostate Cancer

The most clearly established risk factors for PrCa are increased age, ethnicity and family history of PrCa and certain other cancers. Familial aggregation of PrCa is one of the strongest risk factors, and provided support for the likely existence of genetic risk factors shared among families. Men with one male first-degree relative (FDR; father or brother) diagnosed with PrCa themselves have an estimated relative risk of approximately 2.5, and risk of diagnosis with PrCa further increases for men with multiple affected FDRs and lower ages at their diagnoses [13,14,15]. Research comparing mono- and dizygotic twin pairs subsequently provided strong evidence for a substantial heritable component in PrCa development [16], believed to be higher than for any other form of common cancer, with the latest estimate of PrCa heritability at 58% and the role of genetic factors consistently high across age groups [17].

Evidence has also been presented for clustering of aggressive clinical presentation of PrCa within families, suggestive for heritability of aggressive PrCa phenotypes. A number of studies from the Swedish population have demonstrated increased risk of high grade PrCa observed among brothers of cases with high grade disease [18], with greater concordance of high risk disease between monozygotic twins [18], familial clustering of fatal PrCa [19], and concordance of good or short PrCa specific survival times between affected fathers and sons [20,21]. A large study that calculated relative risks for lethal PrCa in the United States based on the number of affected FDRs estimated increased relative risks ranging from 2.49 for males with 1 affected FDR to 5.30 for ≥3 affected FDRs, with higher risk also observed for greater numbers of affected second- and third-degree relatives [22]. In the Swedish population, absolute risk of PrCa was calculated to be 12.9% by age 75 and 5.2% for high-risk disease; with these estimates rising with increasing family history of PrCa to 30.3% and 8.9%, respectively, for men with one affected brother, and to 63.6% and 20.5% for men with two affected brothers and an affected father [23].

PrCa also clusters in families with a strong family history of other cancer types, especially breast and ovarian cancer [24,25,26], whilst PrCa is now also widely regarded as among the Lynch syndrome spectrum of cancers [27,28,29,30]. Hereditary breast and ovarian cancer syndrome is frequently associated with germline mutations in the *BRCA1* and *BRCA2* genes, whilst Lynch syndrome is categorised by germline mutations in DNA mismatch repair genes. A strong family history of PrCa remains a more effective indicator of PrCa risk than family history of other cancer types however, especially with respect to early-onset and lethal disease [31].

PrCa incidence and mortality rates differ substantially across ethnic groups, with the greatest burden of PrCa and highest mortality rates afflicting men of African ancestral origin and lowest observed in men of Asian ancestry [32]. In the United States, PrCa incidence rates are estimated to be approximately 1.76-fold higher in African American men than Caucasians, with relative mortality rates from PrCa elevated 2.20-fold among black men [33]. Whilst these disparities may in part result from socioeconomic, cultural and healthcare availability or treatment preference factors, differences in predisposition towards PrCa development arising through varying allelic frequencies of heritable genetic risk factors between ancestral groups are also likely [34]. Differences in PrCa incidence rates are also observed between men of the same race and ethnicity residing in different countries however, suggesting that these also occur in part due to environmental factors [32]. 

Age-specific PrCa incidence rates rise steeply after age 50, with a peak incidence rate per 100,000 men in the UK between 2015 and 2017 among the 75–79 age group [35]. The incidence of younger onset PrCa (aged ≤55 years) is however increasing and represents a greater proportion of total new diagnoses [36]. Early-onset PrCa patients generally also demonstrate poorer cause-specific survival than men diagnosed at older age ranges and are likely to represent a cohort enriched for genetic variants that increase susceptibility to PrCa, and which could benefit substantially from well informed screening and genetic testing initiatives [36,37].

## 3. Initial Approaches for the Identification of PrCa Susceptibility Genes

Substantial advances in the technologies available for application in genomics research have been made during the past quarter of a century, especially since the year 2000. Prior to these developments however, many early efforts to identify PrCa susceptibility genes revolved around genetic association studies examining biologically plausible candidate genes [38]. These approaches demonstrated modest success, although were frequently limited by factors including sample size constraints inherent to the available lower-throughput techniques of the era, population stratification, the genotyping only of specific founder mutations within a cohort rather than screening of full coding sequences of genes, and publication bias against the reporting of negative results. The CAG and GGN trinucleotide repeat polymorphisms within the transactivation domain of the androgen receptor gene are one example of an extensively studied candidate, however evidence for association between repeat length and PrCa risk has been inconsistent and modest [39,40,41,42,43,44]. Several other variants, genes or pathways were also examined under this approach, including the androgen [45] and oestrogen [46] metabolic pathways, and *TP53* gene [47]; again with inconsistent evidence of association and magnitude or direction of effect frequently reported between studies. Another widely investigated class of candidate was DNA repair genes, for which associations with overall, aggressive or young onset disease have been reported for a handful of genes including *BRCA2* [48,49,50,51,52], *BRCA1* [53,54,55], *CHEK2* [56], and *NBN* [57,58].

Techniques such as genetic linkage or admixture analyses also permitted genome level scans and therefore did not require *a priori* hypotheses of the identity or location of candidate PrCa susceptibility genes. A seminal example of the isolation of a disease gene through linkage methods in a common, complex disease was the mapping in 1990 of a major breast and ovarian cancer predisposition locus to Chr17q21 [59,60], later traced to the *BRCA1* gene using positional cloning [61,62]. Linkage studies for non-Mendelian traits in humans are an arduous process, seeking to identify shared stretches of DNA between genetic markers that cosegregate among affected individuals within large family units in which multiple cases of a particular disease cluster [63,64]. Statistically significant linkage intervals may also be long and contain large numbers of candidate genes. Linkage signals within families may also potentially be obscured due to the occurrence of phenocopies (sporadic occurrence of the disease in individuals that did not inherit the susceptibility allele), or by incomplete and age-dependent penetrance of disease among risk allele carriers; therefore these studies are primarily powered for the detection of high penetrance variants. Linkage analyses for PrCa were typically performed using large families that meet criteria for hereditary prostate cancer [65] and from which multiple PrCa patients and unaffected men were willing to contribute DNA. Pedigrees enriched for clustering of clinically significant or young-onset disease were also prioritised, in order to maximise the prospect of identifying genes influencing poorer prognosis PrCa phenotypes and minimise the likelihood of confounding through instances of sporadic, nonhereditary PrCa. In contrast to genetic linkage analyses, admixture mapping is performed using a study cohort that contains admixture from two or more ancestral populations that experience differences in rate of a phenotype or disease, under the hypothesis that casual variants affecting the trait will occur more frequently on segments of DNA inherited from the higher risk ancestral population [66]. The resolution achieved by admixture mapping approaches is substantially greater than that of linkage studies, reducing the likely number of candidate genes at an identified association.

**Figure 1:**
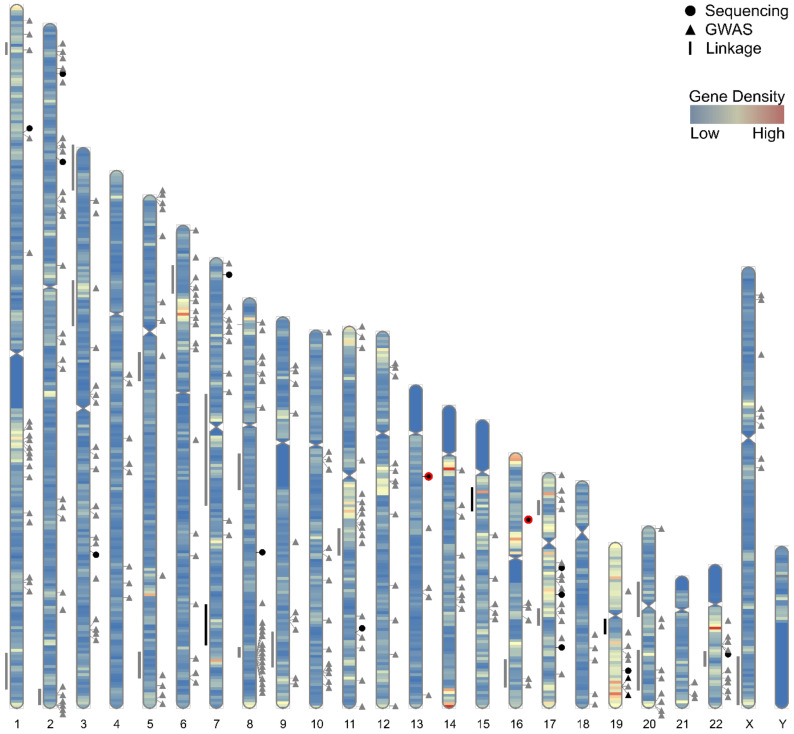
Karyogram depicting the approximate locations of candidate PrCa risk loci reported through linkage, GWAS and sequencing study approaches. To the right of the chromosome ideogram, triangle symbols indicate the position of 269 reported independent GWAS index variants [127] and circle symbols the locations of DNA repair genes reported as a potential candidate for risk in two or more PrCa sequencing studies (listed within Table 1). To the left of the chromosome ideogram, line symbols show the approximate intervals of linkage peaks according to representative markers for the region or cytogenetic band co-ordinates. Grey coloured symbols indicate no or limited evidence for association with risk of aggressive PrCa, the black colour denotes moderate or conflicting evidence for risk of aggressive disease and black symbols with a red border signify stronger evidence for a contribution towards poorer prognosis phenotypes. The karyogram is overlaid with a heat map depicting gene density across the human genome in 1 Mb windows and was generated using RIdeogram [128] and custom plotting functions.

The first putative high risk PrCa locus identified through linkage, located at Chr1q24-25 and denoted HPC1, was reported in 1996 [67]. This signal was observed primarily within families with multiple early-onset cases [68] and subsequently assigned to the *RNASEL* gene [69]; however, replication of the HPC1 locus in independent cohorts has proven inconsistent [70,71,72,73,74,75,76,77]. Subsequent proposed PrCa susceptibility loci reported in linkage or admixture mapping analyses include Chr1p36 [78,79,80], Chr1q42.2-43 [73,81,82], Chr2q37.3 [83,84,85], Chr3p14 [86], Chr3p25-26 [87,88], Chr5q11-12 [76,89], Chr5q35 [76,89,90], Chr6p22.3 [89,91], Chr7q11-21 [92], Chr7q31-33 [90,93,94,95], Chr8p22-23 [79,96,97,98], Chr8q12-13 [76,89], Chr8q24 [90,99,100], Chr9q34 [101,102], Chr11q14 [88,89,91], Chr15q11-14 [76,103,104,105], Chr16q23 [84,101], Chr17p12 [106,107,108,109], Chr17q21-22 [83,105,110], Chr19q12-13 [93,111,112,113], Chr20p11-q11 [89,91], Chr20q13 [114,115,116], Chr22q12.3 [76,89,117,118,119,120], and ChrXq27-28 [121,122,123,124] (Figure 1). Several of these loci also demonstrated greater evidence for linkage in families with higher numbers of affected relatives or containing multiple early-onset cases, whilst the loci at Chr7q31-33, Chr15q and Chr19q were reported to associate with risk of more aggressive disease. Evidence from validation studies and gene mapping approaches again proved conflicting for many of these loci however [101,125,126]; therefore, although rare higher penetrance risk alleles at these loci may be present within specific families, at population levels the majority of these loci appear unlikely to account for a large proportion genetic susceptibility to PrCa or aggressive disease.

One notable success for linkage-based analyses in PrCa families, however, was the robust identification of *HOXB13* as the first gene accounting for a substantial fraction of familial PrCa. Evidence for linkage on chromosome 17q was first reported based on 175 pedigrees [110], and subsequently refined to a 10cM interval (approximately 15.5Mb) at Chr17q21-22, containing 202 candidate genes [129]. In a seminal report, Ewing et al. performed targeted sequencing of the coding regions of these genes in 94 unrelated men from hereditary PrCa families; identifying a recurrent nonsynonymous *HOXB13* variant (rs138213197/G84E) in four of these probands. This variant was observed to cosegregate in all 18 PrCa cases with DNA available within these four families and was also enriched among cases in a replication cohort, particularly among men with either or both young-onset PrCa or a family history of PrCa [130]. *HOXB13* G84E was subsequently confirmed as a moderate penetrance PrCa susceptibility variant in European ancestry populations through a number of independent studies, with consistent evidence demonstrated for strong associations with early-onset and familial PrCa, but importantly no evidence for increased risk of poorer prognosis disease in mutation carriers [131,132,133,134,135,136,137,138,139,140]. *HOXB13* G84E mutations are estimated to be present in up to 5% of European ancestry families with hereditary PrCa [140], whilst average estimated risks of developing PrCa for heterozygous male carriers are approximately 17% by age 65 rising to 62% by age 85, and further increasing with stronger family history of PrCa [141]. The *HOXB13* G84E mutation is found almost exclusively in European ancestry populations [131,132,133,140,142]. Other recurrent *HOXB13* mutations have, however, subsequently been observed in Chinese [143] and Japanese [144] men with PrCa, and additional rare nonsynonymous mutations within African ancestry [130] and Portuguese [145] PrCa families, indicating a contribution by germline *HOXB13* mutations in susceptibility to PrCa in diverse populations and ethnic groups.

Besides *HOXB13* however, linkage approaches were largely unsuccessful at definitively isolating higher penetrance PrCa susceptibility genes attributable to a substantial fraction of disease heritability among populations. These outcomes implied that the allelic spectrum of PrCa risk variants could therefore more closely resemble a ‘Common Disease, Common Variant’ model, under which larger numbers of low penetrance but frequently occurring variants are the main contributors to genetic risk of the disease, rather than a ‘Common Disease, Rare Variant’ hypothesis, in which rare, high penetrance variants are the major initiator. Although soon to be superseded by emerging higher throughput approaches for common variant association analyses, linkage and admixture mapping based techniques also successfully identified the first evidence for common variation conferring susceptibility to PrCa, at the Chr8q24 locus [99,100].

## 4. Genome-Wide Association Studies for Common, Low Penetrance PrCa Susceptibility Loci

The completion of the Human Genome Project [146,147] alongside catalogues of common human variation and linkage disequilibrium patterns [148,149] heralded a significant development for research into genetic susceptibility to common diseases. These resources facilitated simultaneous, unbiased and lower cost association testing of large numbers of variants throughout the genome in the form of genome-wide association studies (GWAS) [150]. The construction of SNP-arrays to interrogate large numbers of genetic variants [151], alongside imputation panels and software for the inference of additional nongenotyped variants [152], were accompanied by development of robust methodologies for handling population stratification [153], establishing statistical significance [154,155] and the meta-analysis of data from discrete studies [156]. Although many formative GWAS were underpowered to detect variants with modest effect sizes or minor allele frequencies (MAF) [157], steadily increasing sample sizes have led to the identification of large numbers of phenotype associated loci, the majority of which had not previously been indicated through alternative approaches [158,159] (Figure 1).

The first GWAS conducted for PrCa confirmed the risk locus initially identified through linkage and admixture mapping at Chr8q24, and identified additional independent risk signals within this locus [160,161,162,163,164]. Chr8q24 remains the major locus contributing to PrCa susceptibility [165,166] across ancestral groups [127], with multiple independent risk signals discovered within the locus, including rare or low frequency moderate penetrance susceptibility variants that are population specific or enriched [136,167,168,169]. The Chr8q24 locus is also established as a significant contributor to familial PrCa [167,170,171] and a diverse spectrum of other cancer types [172]. Many of these Chr8q24 risk signals are located a substantial distance from genes, however a number of plausible candidate variants within regulatory elements have been identified [173,174]. These are implicated in regulating the expression of multiple protein coding genes including the *MYC* proto-oncogene [175,176,177,178], *POU5F1B* transcriptional activator [165,179] and *FAM84B* gene [113,180], and long noncoding RNAs [181] including *PVT1* [178,182], *PCAT1* [178,183] and *PRNCR1* [178,184]. This indicates that PrCa risk modulation by the Chr8q24 locus is likely to be influenced through a diverse and complex range of biological mechanisms.

A number of subsequent European ancestry GWAS and meta-analyses of increasingly large sample sizes have reported rapidly expanding numbers of loci associated with PrCa risk outside of the Chr8q24 region, many of which loci also contained multiple independently associated risk signals [185,186,187,188,189,190,191,192,193,194]. As larger sample sizes are employed, the novel loci identified generally exhibit diminishing index variant effect size and/or MAF, however, are identified in greater numbers. Several of these PrCa risk loci contain genes heavily linked to prostate function, or whose expression is enriched in the prostate relative to other tissues [195]; however, the biological mechanisms underpinning the majority of PrCa risk loci identified through GWAS generally remain poorly characterised at present. Functional validation of a small number of candidate causal variants has been performed to date [169,175,178,196,197,198,199,200,201,202,203,204,205], whilst fine-mapping of association signals and in silico annotation procedures have also helped to narrow the pool of likely candidate causal variants and identify prospective target genes and biological mechanisms that may give rise to differential risk [173,206,207,208,209]. A related methodology, transcriptome wide association studies (TWAS), in which GWAS summary statistic datasets and SNP-gene expression data from a reference panel are integrated, for the purpose of imputing gene expression data into phenotyped datasets which lack directly measured expression data, has also been employed in order to identify prospective gene–trait associations and prioritise putative candidate genes at many GWAS loci [210]. For PrCa, individual large TWAS have so far reported between 38 and 217 genes significantly associated with PrCa risk [211,212,213], including a number of the most strongly implicated functional candidates identified through other approaches in addition to candidate genes not previously proposed. PrCa TWAS have also identified a handful of additional novel candidate PrCa predisposition regions and corresponding candidate genes that had not previously been implicated in disease risk through significant GWAS associations [211,212].

**Figure 2:**
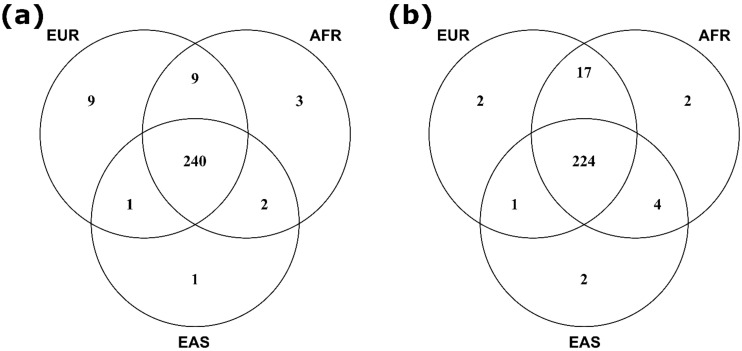
Venn diagrams comparing the proportion of the 269 independent GWAS index variants reported to date that were (**a**) present at ≥1% risk allele frequency, and (**b**) present at ≥5% risk allele frequency in European (EUR), African (AFR) and East Asian (EAS) ancestral super-populations.

The majority of samples included in published PrCa GWAS to date are of European ancestry; however, a number of studies have also been conducted for men of African [214,215,216,217], Japanese [180,218,219,220], Chinese [220,221], and Latino [222] ancestries. Despite currently unequal power across ancestral populations studied, many susceptibility loci reported as statistically significant in large European ancestry GWAS have also replicated at genome-wide significance in additional populations, whilst further risk signals specific to or that have substantially enriched risk allele frequency within non-European ancestral populations have also been identified. The remaining loci discovered in European ancestry GWAS but which have not yet formally validated across other ancestries have also generally demonstrated consistent directionality of effect in additional populations [223,224,225], indicating a strong likelihood that common functional causal alleles shared across multiple populations at varying allelic frequencies will underlie the majority of GWAS loci reported at this point in time. These observations facilitated the aggregation of samples from multiple ancestries to perform larger multiethnic meta-analyses, which identified additional cross-ancestry risk loci not detectable through existing sample cohort sizes from any individual ancestral population [194]. The largest meta-analysis for PrCa risk so far conducted, comprising a total of 107,247 cases and 127,006 controls from European, African, Asian, and Hispanic ancestry populations, although predominantly European ancestry men, has reported evidence for 269 independent PrCa susceptibility signals (Figure 1), of which 183 had been identified through previous GWAS [127]. These 269 PrCa risk signals are situated within 176 distinct genomic loci when defined as >800kb from any neighbouring independently associated index variant, and the overwhelming majority of index variants are common, with their risk allele frequencies ≥5% in multiple ancestral populations (Figure 2).

A recent report estimated the genetic architecture of PrCa to be modulated by approximately 4500 common susceptibility variants, of which a greater proportion of risk variants confer larger effect sizes than observed for other cancer sites with comparably powered GWAS available [226]. This analysis indicates that large numbers of PrCa risk loci potentially remain to be discovered; albeit predominantly with diminishing per variant effect sizes or risk allele frequencies. The same simulation also estimated that approximately 250,000 cases and an equal number of controls would be required for the identification of variants explaining approximately 70% of the GWAS heritability at the standard genome-wide significance threshold, and 500,000 of each cohort to achieve 80% of heritability [226]. According to heritability estimates, this study also estimated a maximum theoretical achievable relative risk due to common variation for a man in the top risk percentile of approximately 5-fold greater than a man at average risk [226]; a relative risk level comparable to carriers of many monogenic susceptibility mutations for diseases, but substantially more prevalent within the general population [227].

Although GWAS have identified a large number of loci associated with risk of developing PrCa and shed light on the biological underpinnings of disease development, the ability of these loci to inform clinical management pathways remains unclear. In particular, whether a specific subset of loci or the cumulative polygenic burden of inheriting greater numbers of risk loci are predictive for risk of developing aggressive disease. A number of studies have reported potential susceptibility loci for aggressive disease based on analyses of cases with aggressive PrCa versus controls [161,228,229,230,231,232,233,234]; however, these comparisons do not definitively demonstrate that a risk locus is associated specifically with the aggressive disease state rather than all cancers. Indeed, case-only analyses comparing PrCa patients with aggressive and low risk phenotypes have so far been unable to provide support for a widespread ability of common risk loci identified in GWAS for overall PrCa to discriminate between patient outcomes [235,236,237].

Case-only GWAS have to date reported at a genome-wide significant threshold two loci associated with Gleason score [238], one with more aggressive phenotype [239] and another with shorter PrCa-specific survival [240]. These loci have not separately associated with risk of overall PrCa through case–control GWAS and could therefore represent disease state specific prognostic markers warranting further investigation. The *AOX1* gene locus associated with PrCa survival time is particularly noteworthy, as the index variant also associated with *AOX1* expression and *AOX1* levels in turn with biochemical recurrence of PrCa [240], whilst the methylation status of this gene had previously been suggested as a candidate biomarker for PrCa outcomes [241,242]. These prospective loci for risk of aggressive disease however remain to be confirmed in independent studies and ancestral populations, and their clinical utility needs to be fully established. A large case-only PrCa GWAS excluding patients with intermediate disease aggressiveness observed evidence for association between aggressive status and variation at the *KLK3* locus, however, no other common variants were associated, including those previously reported with respect to overall disease or phenotypic indicators of poorer prognosis [Saunders et al., Manuscript in preparation]. This is consistent with previous reports that the *KLK3* GWAS SNP rs2735839 is associated with Gleason score in addition to overall disease risk [243,244,245,246,247]. In all of these studies, the risk allele of rs2735839 for overall PrCa was however overrepresented in patients with nonaggressive disease and thus inversely associated with aggressive status; leading to caution that the associations observed may relate to detection bias of indolent disease due to raised PSA expression in carriers of this genotype [246,247]. In independent study cohorts, the association between *KLK3* and less aggressive disease was however not substantially attenuated by adjusting for PSA levels at diagnosis [Saunders et al., Manuscript in preparation] or remained associated with aggressive status among only patients with low PSA levels from two separate ancestral populations [248]. The full role of *KLK3* variation in PrCa susceptibility, risk of aggressive disease and serum PSA levels may therefore warrant more extensive investigation, especially in prospective or PSA naïve cohorts, and may have the potential to enable the identification of a subset of individuals at lower risk of poor prognosis disease who could benefit from less interventionist treatment options.

Whilst these initial case-only reports imply that caution is warranted regarding the potential for GWAS loci to be able to accurately stratify PrCa patients more likely to develop clinically significant disease, increasing evidence supports the improving ability of genetic risk scores (GRS; also frequently referred to as polygenic risk scores/PRS) incorporating ever larger numbers of established susceptibility variants to identify a population subset at greater lifetime risk of diagnosis with PrCa of any severity [127,249,250,251,252,253,254,255,256,257]. This suggests the prospect that future PrCa screening programs could be targeted towards only a specific segment of the population at the greatest risk, to facilitate earlier identification of the majority of patients who will progress to develop poorer prognosis disease whilst potentially concurrently reducing levels of overdiagnosis of men with indolent disease [258,259,260]. GRS for complex common diseases comprise the sum of the germline risk alleles for the disease that an individual possesses, weighted by the effect estimates for each risk variant, and are used to estimate the individual’s lifetime risk for developing the disease [261]. GRS have to date primarily been developed using risk variant catalogues and effect estimates compiled from large European ancestry GWAS discovery populations, and perform less optimally when applied to populations with divergent ancestry [262]. The use of trans-ethnic variant discovery and effect size estimation approaches has, however, demonstrated promise for improving cross-population risk prediction performance, with the latest 269 variant PrCa GRS established through a multiethnic meta-analysis framework reporting a mean GRS 2.18-fold higher for men of African ancestry and 0.73-fold lower for men of East Asian ancestry in comparison to men of European ancestry [127].

Although the ability of GRS to predict disease status within cohort studies has been demonstrated for various traits, their potential clinical utility to inform screening decisions for individual members of the population does however largely remain to be established at this point in time. Their potential consideration for implementation as a prospective risk-profiling tool prior to screening for PrCa may, however, soon become warranted. A polygenic hazard score (PHS) has demonstrated initial promise for the detection of clinically significant PrCa at younger age among higher PHS percentiles [263,264], including in men from diverse ancestral groups [265]; however, the PHS associated with PrCa of any severity and was not able to differentiate specifically for the subset of men who develop clinically significant disease. Coupled with advances in prostate imaging techniques, approaches of this nature do, however, hold promise for facilitating personalised, genetically informed screening decisions to enable early detection of cancers in men at higher risk of developing PrCa of any severity, to be followed by the application of appropriate treatment decisions after diagnosis. However, whilst initial studies to assess the feasibility of genetics-informed screening approaches at the population level have been undertaken, further evaluation and refinements are likely to be required before GRS can be widely integrated into public healthcare systems; particularly in respect to equitable applicability across diverse ethnicities and ascertaining appropriate thresholds for benefit–harm trade off and cost effectiveness.

## 5. Sequencing Studies for Rare, Moderate Penetrance PrCa Susceptibility Genes

Although many common loci have been identified which contribute substantially towards PrCa risk, rare variation is also estimated to play an important role in PrCa heritability [266], especially in men of African ancestry [267]. A number of next-generation sequencing studies reporting rare germline mutation findings in PrCa patients have been conducted in recent years. However, the overall number of samples included in these studies to date remains a fraction of those in GWAS, whilst due to the low allelic frequency of variation primarily examined, large sample sizes are required to achieve sufficient statistical power for moderate penetrance variant detection in complex diseases [268]. To increase power, most sequencing studies, therefore, primarily examine rare protein altering variant frequencies collapsed at the gene or gene-set rather than individual variant level, for which a variety of statistical methodologies have been developed [269]. Many studies also incorporate an extreme phenotype sampling strategy in order to maximise power with limited available sample size [270]. For PrCa, many sequencing studies to date reporting prospective susceptibility genes have taken the form of tumour-sequencing studies that also reported germline findings from matched normal DNA, which were conducted on case cohorts numbering in the hundreds, contained no control cohort and usually specifically examined metastatic castrate resistant (mCRPC) PrCa cases [271,272,273,274,275]. Other similarly sized studies examining germline DNA exclusively have reported mutation frequencies for overall [276,277] and familial [278,279] PrCa, men with PrCa alongside additional primary tumour types [280], or compared rates between aggressive and nonaggressive phenotypes [281,282]. More recently, larger association studies comparing mutation frequencies among cohorts of a few thousand samples have also begun to be completed, predominantly interrogating the coding regions of panels of prospective candidate genes [144,283,284,285]. To date, published PrCa sequencing studies have, however, predominantly either sequenced, analysed or reported only findings primarily related to DNA repair genes, and therefore these remain at present the only class of gene widely scrutinised for rare germline variation in the PrCa setting and for which findings for individual genes may be comparable across a number of separate studies and cohorts.

Sequencing studies of PrCa patients with aggressive disease have consistently reported elevated mutation rates for the *BRCA2* gene, confirming earlier observations linking *BRCA2* mutation carriers to more aggressive phenotypes through other approaches [286,287,288,289,290,291]. Several additional DNA repair genes have also been implicated in multiple sequencing studies as prospective moderate penetrance PrCa susceptibility genes warranting further investigation in larger sample cohorts, especially *ATM*, *BRCA1* and *PALB2* (Table 1, Figure 1). Although these genes are widely included in PrCa sequencing panels in both research and clinical settings, their contribution towards PrCa susceptibility and risk of aggressive disease await definitive confirmation, whilst additional genes associated with risk may remain to be identified through larger study sizes and broader sequencing panels. A recent case-only study comprising 2770 aggressive and 2775 nonaggressive PrCa cases reported statistically significant evidence for substantially increased risk of aggressive disease among germline *BRCA2* and *PALB2* mutation carriers, with *ATM* also nominally associated [284], corresponding with observations of high combined germline and somatic mutation frequencies for these genes among mCRPC patients [292]. Pathogenic germline *PALB2* mutations were present at a far lower rate than *BRCA2* in this study cohort, however, were substantially more enriched among aggressive, metastatic and lethal cases [284]. A number of other studies have also linked *ATM* mutations to poorer prognosis PrCa phenotypes [281,282,293,294]; although the largest retrospective *ATM* sequencing study to date comprising 5560 cases and 3353 controls of European ancestry observed only limited support for association with aggressive disease but strong evidence for increased risk of overall PrCa among *ATM* mutation carriers [295].

PrCa sequencing studies reported to date have primarily been performed using men of European ancestry; however, large panel sequencing studies have also been conducted in African ancestry [285] and Japanese populations [144]. These studies have provided supporting evidence for pan-ethnic contributions towards PrCa risk for particular genes, especially *ATM* and *BRCA2*; however, ethnic specific differences in mutation carrier rates at individual genes were also observed, indicating that the allelic frequency spectrum of moderate penetrance PrCa risk genes could differ substantially across ancestral groups [297]. A sequencing study of aggressive PrCa cases and disease-free controls also implicated rare variants in the *TET2* gene, a locus previously associated with overall PrCa through GWAS [185], as a prospective susceptibility gene for aggressiveness in African American men, with 24.4% of aggressive cases and only 9.6% of controls carrying a rare deleterious *TET2* variant [298]. However, the association between deleterious germline variation and aggressive disease was not observed in the European ancestry cohort of the reporting study and remains to be validated in external cohorts.

Whilst the PrCa sequencing studies conducted to date have achieved some degree of success in identifying a small number of genes linked to substantially increased risks of PrCa onset or poorer prognosis disease phenotypes, and a handful of additional candidates warranting further investigation, the majority of studies were not designed or sufficiently powered to provide accurate risk estimates for individual genes. A recent prospective study of PrCa risk for male *BRCA1* and *BRCA2* carriers estimated standardised incidence ratios of 2.35 and 4.45, respectively, in addition to a stronger association with higher Gleason score and a standardised mortality rate of 3.85 for *BRCA2* carriers [299]. A meta-analysis of mutation data for *ATM* has also quantified likely pathogenic variation in this gene as a moderate penetrance contributor towards PrCa susceptibility, with an odds ratio of 4.4; however, evidence of association with aggressive or younger onset disease was less distinct [295]. Larger sequencing studies or meta-analyses interrogating broader panels of genes would be required in the future to clarify the definitive set of genes linked to higher risks of developing PrCa and enable accurate quantification of risk experienced by mutation carriers of specific genes. The growth of national Biobanks may in the future provide convenient and cost effective means to validate results from germline sequencing studies, whilst large aggregate sequencing data resources such as The Genome Aggregation Database (gnomAD) [300] also allow efficient comparison of variant frequencies across populations. Caution should however be exercised with regard to the direct inclusion of external cohorts alongside internally sequenced samples in rare variant association studies, especially their application specifically as external control cohorts for case-only sequencing data, due to the potential for artefactual results arising from differences in sequencing depths, QC procedures or population stratification between datasets. A role for linkage analyses for the interrogation of rare variation within PrCa family units may also re-emerge as whole-genome sequencing becomes more widely adopted [301].

## 6. Translational Potential of Germline PrCa Susceptibility Variation and Conclusions

Evidence exists for a substantial heritable component for overall PrCa risk, and familial clustering of high risk and fatal PrCa phenotypes. A combination of common and rare variants is likely to influence risk of PrCa, with common variants a substantial contributor at the population level and rare variants important within specific families or sub-groups. Current evidence does not support common variants contributing substantially towards risk of aggressive disease individually or cumulatively, however; with this class of variation appearing primarily to be a substantial driver of initiation of tumorigenesis but subsequently having limited influence on progression to aggressive phenotypes. Whilst rare variant studies have to date been underpowered, a handful of DNA repair genes have been implicated as prospective risk factors for predisposition towards development of aggressive phenotypes among PrCa cases who are mutation carriers.

Germline testing for rare moderate penetrance pathogenic variants in specific genes is becoming an increasingly important focus of PrCa management and treatment, with the additional promise of guiding tailored therapeutic interventions appropriate for targeting specific molecular vulnerabilities in individual patients’ tumours. Although few genes have been conclusively identified to date in which rare variants confer higher risk of developing aggressive PrCa, as further larger, cross population sequencing studies and meta-analyses are performed, additional genes for which mutation carriers experience greater risk of aggressive disease and/or more favourable response to particular treatment options are likely to be established [302]. At present, germline testing in relation to PrCa is recommended primarily to inform treatment options or clinical trial eligibility for patients with metastatic or locally advanced disease, screening of PrCa patients or healthy men with a family history suggestive of hereditary PrCa, active surveillance decisions, or for patients with Ashkenazi Jewish ancestry [303,304]. Genes currently widely advocated for definite inclusion or consideration of inclusion in germline PrCa sequencing panels in one or more of these contexts are *ATM, BRCA1*, *BRCA2*, DNA mismatch repair genes involved in Lynch syndrome (especially *MSH2*, however testing of *MLH1*, *MSH6*, *PMS2* and potentially *EPCAM* is also generally advised) and *HOXB13*. The latest National Comprehensive Cancer Network (NCCN) guidelines for germline testing also propose screening *CHEK2* and *PALB2* as part of their minimum recommended predisposition gene panel [304]. The tumour suppressor gene *TP53* plus additional DNA repair genes including *BRIP1* and *NBN* have also been proposed for inclusion on screening panels, but await more definitive evidence to achieve consensus of utility [303]. In patients with metastatic PrCa, among this gene panel for germline screening, *ATM*, *BRCA1*, *BRCA2* and potentially other DNA repair genes may inform response to PARP inhibitors [296,305,306], *BRCA1*, *BRCA2* and other DNA repair genes sensitivity to platinum chemotherapy [307,308], and DNA mismatch repair genes response to anti-PD-1 immunotherapy [309,310]. Investigation of whether carriers of mutations currently considered actionable specifically in the treatment of metastatic PrCa would also benefit from earlier treatment with targeted therapies prior to progression to incurable metastatic phenotypes may, therefore, also be warranted.

Although family history of the disease is usually the main reason for genetic testing of men without a diagnosis of PrCa, an appreciable proportion of PrCa patients without a family history sufficient to meet NCCN guidelines for germline genetic testing have also been demonstrated to carry rare germline putative PrCa susceptibility variants [277]. Even greater numbers of men towards the upper extremity of common variant GRS distributions may also experience similarly elevated levels of risk to carriers of mutations in moderate penetrance genes [226,227]. Identification of germline variation that modulates PrCa risk therefore holds promise for informing targeted screening programs to facilitate earlier identification of tumours. If coupled with screening for genes specifically linked to higher risks of aggressive disease and appropriate treatment options for early stage disease, such as active surveillance, these insights could simultaneously enable improved survival of patients who would progress towards poorer prognosis phenotypes, alongside reductions in overtreatment of men with clinically insignificant disease. At present, no germline testing guidelines incorporate the use of GRS approaches; however, owing to the common nature of the underlying individual variants of which they are composed, at this point in time this class of variation has actually been more rigorously statistically evaluated for association with phenotypic traits in the research setting than has been the case for many genes in which rare variants are expected to cause non-Mendelian diseases. Given the greater number of men who may experience equivalent levels of risk arising through multiple common, low penetrance variants to those men that are carriers of rare moderate penetrance PrCa susceptibility mutations, and the markedly cheaper cost of genotyping common polymorphisms in comparison to sequencing of gene panels to screen for rare pathogenic mutations, further evaluation of whether GRS may now have developed to sufficiently informative levels to warrant incorporation as a consideration within PrCa germline genetic screening guidelines may soon become appropriate.

The majority of samples included in studies investigating risk factors for PrCa to date have been from populations of European ancestry. However, given differing allelic architecture and frequencies of both rare and common variants between populations, in addition to the higher incidence and poorer prognosis of PrCa among men of African descent, reducing under-representation of additional ethnicities in PrCa research remains an unmet requirement in order to ensure applicability of discoveries across populations and pan-ethnic access to healthcare improvements [311,312,313]. Enabling ubiquitous access to germline genetic testing across national and global healthcare systems would however continue to represent a substantial challenge.

## Figures and Tables

**Table 1:** DNA repair genes described as candidate PrCa susceptibility and/or poor prognosis genes in more than one next generation sequencing study.

Gene	Chromosome	Reporting Studies (Reference Number)
*ATM*	11	[144,271,275,276,277,278,281,282,283,284,285,295,296]
*ATR*	3	[271,275,281]
*BRCA1*	17	[271,277,278,282,283]
*BRCA2*	13	[144,271,275,277,278,281,282,283,284,285,296]
*BRIP1*	17	[271,278]
*CHEK2*	22	[271,277,278,283]
*ERCC2*	19	[281,283]
*GEN1*	2	[271,283]
*MSH2*	2	[271,277,283]
*MUTYH*	1	[275,277,278]
*NBN*	8	[271,281,283,285]
*PALB2*	16	[271,277,278,281,284,285]
*PMS2*	7	[271,277,278,281]
*RAD51D*	17	[271,281]
